# Risk factors for missed abortion: retrospective analysis of a single institution’s experience

**DOI:** 10.1186/s12958-022-00987-2

**Published:** 2022-08-09

**Authors:** Wei-Zhen Jiang, Xi-Lin Yang, Jian-Ru Luo

**Affiliations:** 1 Department of Gynaecology and Obstetrics, Chengdu Women’s and Children’s Central Hospital, School of Medicine, University of Electronic Science and Technology of China, No.1617 of Riyue Avenue, Qingyang District, Chengdu, 611731 China; 2 Department of Radiation Oncology, Chengdu Women’s and Children’s Central Hospital, School of Medicine, University of Electronic Science and Technology of China, Chengdu, 611731 China

**Keywords:** First trimester pregnancy, Missed abortion, Gestational sac, Crown-rump length, mGSD-CRL

## Abstract

**Objective:**

To explore the risk factors including the difference between mean gestational sac diameter and crown-rump length for missed abortion.

**Methods:**

Hospitalized patients with missed abortion and patients with continuing pregnancy to the second trimester from Chengdu Women's and Children's Central Hospital from June 2018 to June 2021 were retrospectively analyzed. The best cut-off value for age and difference between mean gestational sac diameter and crown-rump length (mGSD-CRL) were obtained by x-tile software. Univariate and multivariate logistic regression analysis were adopted to identify the possible risk factors for missed abortion.

**Results:**

Age, gravidity, parity, history of cesarean section, history of recurrent abortion (≥ 3 spontaneous abortions), history of ectopic pregnancy and overweight or obesity (BMI > 24 kg/m^2^) were related to missed abortion in univariate analysis. However, only age (≥ 30 vs < 30 years: OR = 1.683, 95%CI = 1.017–2.785, *P* = 0.043, power = 54.4%), BMI (> 24 vs ≤ 24 kg/m^2^: OR = 2.073, 95%CI = 1.056–4.068, *P* = 0.034, power = 81.3%) and mGSD-CRL (> 20.0vs ≤ 11.7 mm: OR = 2.960, 95% CI = 1.397–6.273, *P* = 0.005, power = 98.9%; 11.7 < mGSD-CRL ≤ 20.0vs > 20.0 mm: OR = 0.341, 95%CI = 0.172–0.676, *P* = 0.002, power = 84.8%) were identified as independent risk factors for missed abortion in multivariate analysis.

**Conclusion:**

Patients with age ≥ 30 years, BMI > 24 kg/m^2^ or mGSD-CRL > 20 mm had increasing risk for missed abortion, who should be more closely monitored and facilitated with necessary interventions at first trimester or even before conception to reduce the occurrence of missed abortion to have better clinical outcomes.

**Supplementary Information:**

The online version contains supplementary material available at 10.1186/s12958-022-00987-2.

## Background

Missed abortion was a special type of spontaneous abortion that the embryo or fetus has already died but remained in the uterus for days or weeks and with a closed cervical ostium [1]. Patients might present with or without subtle clinical symptoms such as vaginal bleeding or abdominal pain. Missed abortion, occuring in approximately 8–20% of clinically confirmed intrauterine pregnancies [2], was often confirmed using ultrasonography.

Missed abortion was undoubtedly a huge physical and psychological setback for women with fertility requirements. Therefore, early identification of women at high risk of missed abortion was pivotal, which might aid in providing possible theoretical basis for implementing clinical measures to prevent missed abortion. Previous studies have revealed that Human Chorionic Gonadotropin(HCG), Estradiol(E2), progesterone, gestational sac diameter(GSD), Crown-Rump Length(CRL), fetal heart rate and yolk sac diameter might be predictive for early pregnancy loss [3–5]^.^ In addition, the predictive value of mGSD-CRL for early pregnancy outcome in in vitro fertilization(IVF) treatment has been established [6]. However, most of the current studies have performed univariate analysis to identify the risk factors for early pregnancy loss [3–6].

Therefore, we conducted this study to more comprehensively explore the possible high risk factors relating to developing of missed abortion using multivariate logistic regression analysis, hopefully it could be of great help to identification and intervention.

## Materials and methods

### Data sources

We reviewed patients from Chengdu Women's and Children's Central Hospital from June 2018 to June 2021. Inclusion criteria of missed abortion group were listed as follows: (1) Not more than 12 weeks gestation; (2) Crown-rump length ≥ seven mm without heartbeat or (3) mean sac diameter ≥ 25 mm without embryo or (4) absence of embryo with heartbeat ≥ two weeks after a scan that showed a gestational sac without a yolk sac or (5) absence of embryo with heartbeat ≥ 11 days after a scan that showed a gestational sac with a yolk sac [7]. Exclusion criteria of missed abortion group were listed as follows: (1) Incomplete information; (2) multiple pregnancy. Patients with the following inclusion and exclusion criteria were enrolled as control group: (1) Patients continued pregnancy to the second trimester were included; (2) Incomplete information and multiple pregnancy were excluded. After excluding patients with incomplete information, 307 patients were finally included with 160 patients having missed abortion and 147 with continuing pregnancy to second trimester. Due to the retrospective nature of the study, informed consent was waived, but this study was granted by the ethics committee of Chengdu Women's and Children's Central Hospital and the ethics approval number was B2021(26).

### Collection of data

Patients’ information regarding age, gravidity, parity, history of vaginal delivery, history of cesarean delivery, history of recurrent abortion (≥ 3 spontaneous abortions), history of induced abortion, history of medication abortion, history of midtrimester induction, history of ectopic pregnancy, history of smoking, history of alcohol consumption, history of other uterine operations, mode of conception, BMI, mGSD-CRL not more than 12 weeks with live embryo were collected.

### Statistical analysis

Categorical variables were described as percentages or frequencies and compared using Pearson *χ2* test; continues variables were described as medians with interquartile range (IQR) and compared with *t* test. We identified the cut-off value for age and mGSD-CRL via X-tile software (version 3.6.1; Yale University, New Haven, CT, USA) once maximal chi-square value reached, which was considered to represent the greatest difference in outcomes prediction among the subgroups [8].

Logistic regression was used to determine independent risk factors for missed abortion. Statistically significant variables from univariate logistic regression analysis (*P* < 0.1) were included in the multivariate analysis. Pearson χ2 test, t test and logistic regression were performed using SPSS (version 25.0, SPSS, Chicago, IL, USA), X-tile software was uesed to calculate cut-off value. G*Power Analysis program (version 3.1, The G*Power Team, Belgium) was used for power calculation. A two-tailed *P* < 0.05 was recognized as statistically significant.

## Results

### Study cohort

**Fig. 1:**
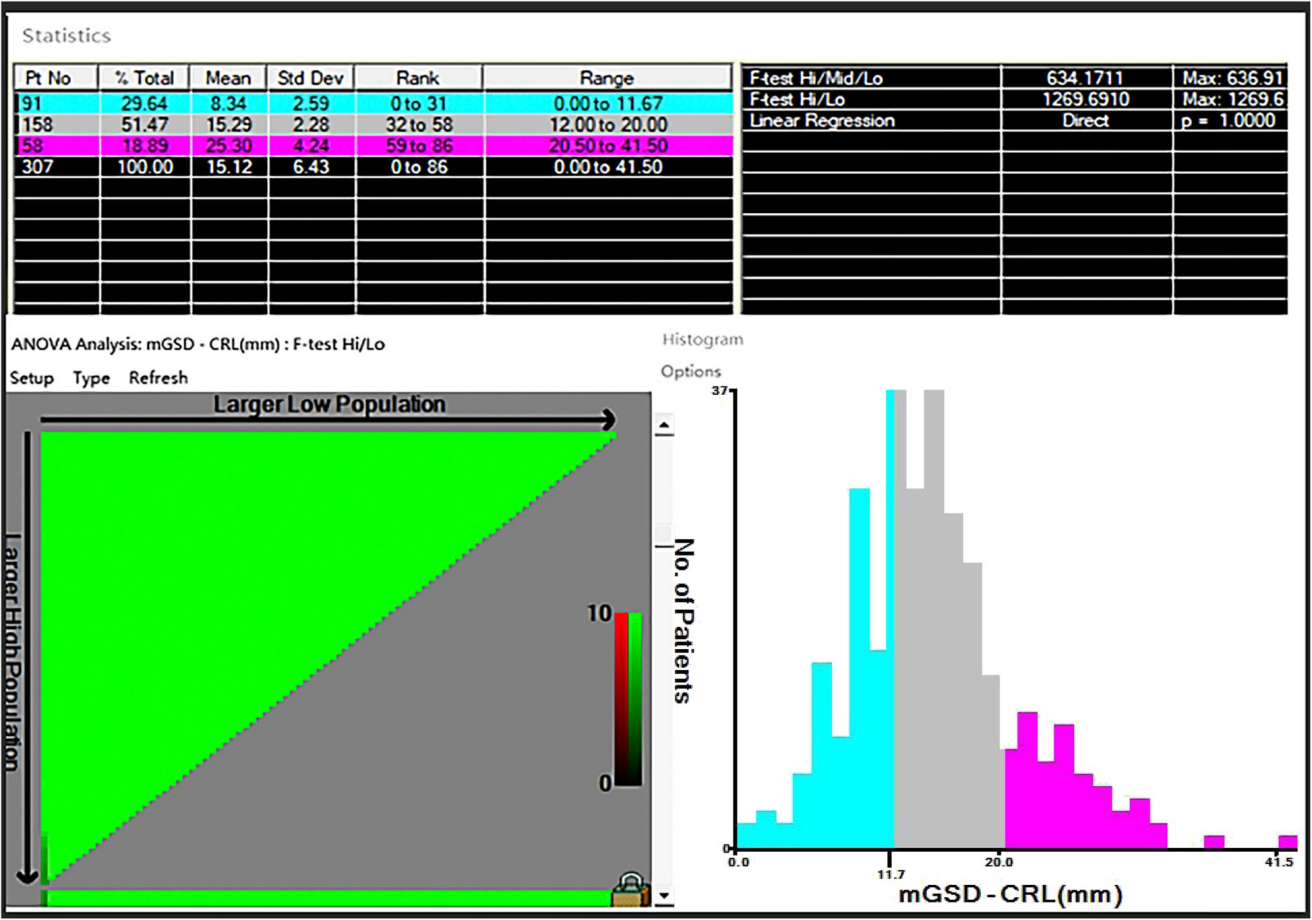
mGSD-CRL at diagnosis stratification by X-tile software

A total of 307 patients were finally included in the study with 160 cases having missed abortion and 147 with continuing pregnancy to second trimester (Supplementary Fig. 1). The characteristics was listed in Supplementary Table 1. As a result, 30 years old was the cut-off value for age via X-tile software. Therefore, age was split as age ≥ 30 years and age < 30 years. Similarly, mGSD-CRL was divided into three subgroups: GSD-CR < 11.7 mm, 11.7 mm ≤ mGSD-CRL ≤ 20.0 mm, GSD-CR > 20.0 mm (Fig. 1). Nearly half of the patients were over 30 years old (49.2%). 38.4% of the patients were having first pregnancy, and the majority of the patients had never delivered (71.0%). 11.1% of the patients had a history of vaginal delivery, however, 18.2% of the patients had a history of cesarean section. Of note, 16% of the patients had a BMI > 24 kg/m^2^, 29.6% of the patients had a mGSD-CRL < 11.7 mm and 18.9% had a mGSD-CRL > 20 mm. Moreover, 2.6% of the patients suffering from recurrent abortion and 4.2% had a history of ectopic pregnancy. Besides, 39.1% of the patients had a history of curettage. In total, 52.1% of the patients developed missed abortion (Table 1).

**Table 1:** The characteristics of risk factors

Variables	Count(%)	Variables	Count(%)
**Age(years)**		**History of medication abortion**	
30	156(50.8%)	Yes	6(2.0%)
≥ 30	151(49.2%)		
**Gravidity(times)**		**History of midtrimester induction**	
1	118(38.4%)	Yes	5(1.6%)
2	79(25.7%)		
3	53(17.3%)	**History of ectopic pregnancy**	
4	36(11.7%)	Yes	13(4.2%)
5	13(4.2%)		
6	7(2.3%)	**Smoking**	
7	1(0.3%)	Yes	1(0.1%)
**Parity(times)**			
0	218(71.0%)	**Alcohol consumption**	
1	83(27.0%)	Yes	1(0.1%)
2	6(2.0%)		
**History of vaginal delivery**		**Other uterine operation**s	
Yes	34(11.1%)	Yes	7(2.3%)
**History of cesarean delivery**		**IVF**	
Yes	56(18.2%)	Yes	9(2.9%)
**History of recurrent abortion**		**BMI(Kg/m**^**2**^**)**	
Yes	8(2.6%)	≤ 24	258(84%)
		> 24	49(16%)
**History of induced abortion**		**mGSD-CRL(mm)**	
Yes	120(39.1%)	< 11.7	91(29.6%)
		11.7 ≤ mGSD-CRL ≤ 20.0	158(51.5%)
		> 20.0	58(18.9%)

### Risk factors for missed abortion

In the univariate logistic regression analysis, Age, gravidity, parity, history of cesarean section, history of recurrent abortion, history of ectopic pregnancy, overweight or obesity (BMI > 24 kg/m^2^) and mGSD-CRL were significantly related to increased risk factors for missed abortion. Furthermore, risk factors identified in the univariate logistic regression analysis were included in the multivariate analysis, which revealed that Age (≥ 30 vs < 30 years: OR = 1.683, 95%CI = 1.017–2.785, *P* = 0.043, power = 54.4%), BMI (> 24 vs ≤ 24 kg/m^2^: OR = 2.073, 95%CI = 1.056–4.068, *P* = 0.034, power = 81.3%), mGSD-CRL (> 20.0vs ≤ 11.7 mm: OR = 2.960, 95% CI = 1.397–6.273, *P* = 0.005, power = 98.9%; 11.7 < mGSD-CRL ≤ 20.0vs > 20.0 mm: OR = 0.341, 95%CI = 0.172–0.676, *P* = 0.002, power = 84.8%) were independent risk factors for missed abortion (Table 2).

**Table 2:** Logistic regression analysis

Variables	OR(95%CI)	*P*-value	OR(95%CI)	*P*-value
	univariate logistic regression analysis		multivariate logistic regression analysis	
**Age(years)**				
30	Reference		Reference	
≥ 30	2.018(1.280–3.181)	**0.002**	1.683(1.017–2.785)	**0.043**
**Gravidity(times)**				
1	Reference			
2	1.009(0.570–1.786)	0.975		
3	1.384(0.722–2.653)	0.328		
4	2.291(1.048–5.006)	**0.038**		
5	2.577(0.752–8.836)	0.132		
6	2.864(0.534–15.353)	0.219		
7	1,850,453,001.811(-)	1.000		
**Parity(times)**				
0	Reference			
1	2.079(1.231–3.510)	**0.006**		
2	1.117(0.220–5.654)	0.894		
**History of cesarean delivery**				
No	Reference			
Yes	2.232(1.209–4.121)	**0.010**		
**Recurrent abortion**				
No	Reference			
Yes	6.680(0.812–54.960)	**0.077**		
**History of ectopic pregnancy**				
No	Reference			
Yes	3.200(0.863–11.863)	**0.082**		
**BMI(Kg/m**^**2**^**)**				
≤ 24	Reference		Reference	
24	1.912(1.011–3.614)	**0.046**	2.073(1.056–4.068)	**0.034**
**mGSD-CRL(mm)**				
11.7	Reference		Reference	
11.7 ≤ mGSD-CRL ≤ 20.0	0.941(0.561–1.577)	0.818	1.009(0.579–1.758)	0.976
20.0	2.804(1.382–5.689)	**0.004**	2.960(1.397–6.273)	**0.005**
20.0			Reference	
11.7			0.338(0.159–0.716)	**0.005**
11.7- 20.0			0.341(0.172–0.676)	**0.002**

## Discussion

Missed abortion, normally presenting without symptoms of threatened abortion such as abdominal pain and vaginal bleeding, was a kind of spontaneous abortion, which were frequently diagnosed using ultrasonography. In this study, we retrospectively analyzed the data of 160 missed abortion patients and 147 pregnant women who didn’t have abortion in the first trimester in order to fully establish the possible risk factors for missed abortion, and provide evidence for early identification and intervention for patients with high risk of missed abortion.

In previous studies, it was believed that advanced age was a high risk factor for missed abortion, which might result from the decline of ovarian function and corpus luteum function as age accrued [1, 9]. However, previous study also showed that advanced age was not a high risk factor for spontaneous abortion [10], in which age was divided into advanced age group (> 35 years) and non-advanced age group (≤ 35 years old). Therefore, we hypothesized that there might be a more meaningful cutoff value other than 35 years old to divide the age into two subgroups. As a result, 30 years old, calculated via x-tile, showed significant value in the final multivariate logistic analysis (OR = 1.683, 95%CI = 1.017–2.785, P = 0.043). As controversial regarding age existed in previous studies, our result showing that age > 30 was an independent risk factor for missed abortion seemed solid. And the dropping from 35 to 30 in terms of cut-off value for age might be related to factors like increasing pressure, unhealthy living habits and environmental pollution resulting from social developing [2, 11]. Although the cut-off value in our study were not consistent with previous ones, the consensus on older age was a high risk factor for missed abortion was basically reached.

A meta-analysis including 16 studies demonstrated that BMI > 25 kg/m^2^ was a high risk factor for abortion [12], which reported that the missed abortion rate of overweight or obese women was as high as 25–37% [13]. The participants from our study were childbearing age women from China, so the definition of overweight or obese as BMI > 24 kg/m^2^ was used for grouping though the World Health Organization(WHO) defined overweight or obesity as BMI > 25 kg/m^2^ [14]. And the result showed that patients with BMI > 24 kg/m^2^ were more likely to have missed abortion than BMI ≤ 24 kg/m^2^ (OR = 2.073, 95% CI = 1.056–4.068, P = 0.034), which was consistent with previous studies [11, 12]. Therefore, weight control before pregnancy was usually recommended.

Although the effect of mGSD and CRL on missed abortion had been reported [3, 4, 15–18], there was few studies working on the relationship between mGSD-CRL and missed abortion. Bromley et al.firstly proposed the concept of "small gestational sac" [19]. And their work revealed that mGSD-CRL < 5 mm in the first trimester was a high risk factor for missed abortion. However, the extremely limited number of included patients in their study might impede the generalization of the conclusion. Similarly, the research from Kapfhamer el also showed that mGSD-CRL < 5 mm was a high risk factor for early pregnancy loss, and further demonstrated that mGSD-CRL > 10 mm was a protective factor for early pregnancy loss [6]. However, Zhao et al. believed that "large gestational sac"(mGSD-CRL ≥ 18 mm) was related to increasing risk for spontaneous abortion [20]. Therefore, we used x-tile to find the two optimal cutoff values for mGSD-CRL, which showed that patients with mGSD-CRL > 20 mm was more more likely to have missed abortion than patients with mGSD-CR ≤ 20 mm. And there was no statistical difference between mGSD-CRL < 11.7 mm group and 11.7 ≤ mGSD-CRL ≤ 20.0 mm group. In summary, we were inclined to believe that increasing mGSD-CRL was associated with increasing risk of missed abortion, which should be further validated in the future due to the differences in sample size from previous studies [6, 19, 20].

Age, gravidity, parity, history of cesarean section, history of recurrent abortion, history of ectopic pregnancy, BMI and mGSD-CRL were identified in the univariate analysis. However, only age, BMI and mGSD-CRL were still meaningful in multivariate analysis. What was inconsistent with previous studies in our study was that recurrent abortion was not a high risk factor for missed abortion [21], which might result from the low incidence of recurrent abortion (missed abortion group vs non-missed abortion group: 7 vs 1) in our study.

One major strength of this study was that stratifying age by x-tile rather than 35 years were firstly recognized for high risk of missed abortion. Other strengths included that mGSD-CRL were analyzed instead of mGSD or CRL independently. On the contrary, This study was inevitably limited by the retrospective nature. In addition, the pathogenic factors for missed abortion was complicated, and some possible high risk factors like immunological or genetic factors could not be obtained.

It is well known that missed abortion is a special type of spontaneous abortion and the ultimate outcome is embryonic arrest. The current knowledge of the missed abortion mostly relates to prevention and treatment, but the classification and severity have not been covered yet according to existing literature and guidelines. The purpose of this paper is to explore the high-risk factors of missed abortion, therefore treatment was barely involved, and we will do more research on the treatment of missed abortion in future work. Overall, We hope that the present study could aid in abortion prediction and treatment decision-making for clinicians.

## Conclusions

This study demonstrated that age ≥ 30 years old, BMI > 24 kg/m^2^ and mGSD-CRL > 20 mm were independent risk factors for missed abortion. This study provided a theoretical basis for clinicians to deliver prompt interventions in childbearing age women during the first trimester or even before pregnancy, so as to reduce the incidence of missed abortion.

## Supplementary Information

**Additional file 1: Supplementary Figure 1.** Flow chart depicting for inclusion of studysubjects. **Supplementary Table1. ** Clinical characteristicsof participants.

## Data Availability

All data that support the findings of this study were available from the corresponding author via E-mail due to appropriate request.
