# Supplementary material for: Risk factors for missed abortion: retrospective analysis of a single institution’s experience
Source: Reprod Biol Endocrinol. 2022 Aug 9;20:115. doi: 10.1186/s12958-022-00987-2 (PMC9361553; doi:10.1186/s12958-022-00987-2)
Supplement: Supplementary file 1 — Additional file 1: Supplementary Figure 1. Flow chart depicting for inclusion of studysubjects. Supplementary Table1. Clinical characteristicsof participants. [file 12958_2022_987_MOESM1_ESM.doc]

**Supplementary materials**

**

**

**Supplementary Figure.1** Flow chart depicting for inclusion of study subjects.

**Supplementary table.1** Clinical characteristics of participants

| **Variables** | **Missed abortion** | **Control group** | **P-value** |
| --- | --- | --- | --- |
| Age < 30 | 68(42.5%) | 88(59.9%) | 0.002 |
| Age > 30 | 92(57.5.%) | 59(40.1%) |
| **Gravidity(times)** |  |  |  |
| 1 | 55(34.4%) | 63(42.9%) | 0.173 |
| 2 | 37(23.1%) | 42(28.6%) |
| 3 | 29(18.1%) | 24(16.3%) |
| 4 | 24(15.0%) | 12(8.2%) |
| 5 | 9(5.6%) | 4(2.7%) |
| 6 | 5(3.1%) | 2(1.4%) |
| 7 | 1(0.6%) | 0 |
| **Parity(times)** |  |  |  |
| 0 | 103(64.4%) | 115(78.2%) | 0.022 |
| 1 | 54(33.8%) | 29(19.7%) |
| 2 | 3(1.9%) | 3(2.0%) |
| **History of vaginal delivery** | 19(11.9%) | 15(10.2%) | 0.641 |
| **History of cesarean delivery** | 38(23.8%) | 18(12.2%) | 0.009 |
| **History of recurrent abortion** | 7(4.4%) | 1(0.7%) | 0.042 |
| **History of induced abortion** | 64(40.4%) | 56(38.1%) | 0.733 |
| **History of medication abortion** | 4(2.5%) | 2(1.4%) | 0.471 |
| **History of midtrimester induction** | 4(2.5%) | 1(0.7%) | 0.208 |
| **History of ectopic pregnancy** | 10(6.3%) | 3(2%) | 0.067 |
| **Smoking** | 1(0.6%) | 0 | 0.337 |
| **Alcohol consumption** | 1(0.6%) | 0 | 0.337 |
| **Other uterine operation**s | 5(3.1%) | 2(1.4%) | 0.301 |
| **IVF** | 3(1.9%) | 6(4.1%) | 0.252 |
| **BMI(Kg/m2)** |  |  |  |
| **≤24** | 128(80%) | 130(88.4%) | 0.044 |
| **>24** | 32(20%) | 17(11.6%) |
| **mGSD-CRL(mm)** |  |  |  |
| **<11.7** | 44(27.5%) | 47(32%) | 0.003 |
| **11.7≤ mGSD-CRL<=20.0** | 74(46.3%) | 84(57.1%) |
| **>20.0(mm)** | 42(26.3%) | 16(10.9%) |
